# Placing problematic media use in context: a research synthesis, person-centric framework, and chart review among a clinical sample of US youth

**DOI:** 10.3389/fpsyt.2025.1574502

**Published:** 2025-09-30

**Authors:** Michael C. Carter, Nicole Powell, Benoit Bediou, Michael Tsappis, David Bickham, Michael Rich

**Affiliations:** ^1^ Digital Wellness Lab, Boston Children's Hospital, Harvard Medical School, Boston, MA, United States; ^2^ Department of Pediatrics, Harvard Medical School, Boston, MA, United States; ^3^ Division of Adolescent/Young Adult Medicine, Boston Children's Hospital, Harvard Medical School, Boston, MA, United States; ^4^ Department of Psychology, University of Geneva, Geneva, Switzerland; ^5^ Department of Psychiatry, Harvard Medical School, Boston, MA, United States

**Keywords:** gaming disorder, internet gaming disorder, problematic use of internet, problematic social media use, information binging, pornography use

## Abstract

Understanding the dynamics underlying problematic media use (PMU) is crucial in today’s digital society. The maintaining factors driving problematic use span both bio-psychological and social factors, necessitating the development of an integrative, meta-theoretical account of PMU to encompass core pathways across established frameworks. The present study used a mixed-methods approach to analyze patient charts (*N* = 205) from a US clinic specializing in addressing PMU. In doing so, we developed the Person–Context–Process–Outcome–Time (PC-POT) model. PC-POT approaches PMU as a cycle of media-dependent dysfunction. Results suggested that this cycle compounds in severity over time and is maintained by a set of (intrapersonal and interpersonal) situational transitions that can affect patient functioning across five key domains. By providing a heuristic structure that more holistically encompasses core determinants and outcomes of PMU, PC-POT helps to provide a more unified basis to advance understanding of PMU in a person- and process-centric way.

## Introduction

A 13-year-old girl, EN, has attention-deficit-hyperactivity disorder (ADHD). She admits that she uses media a lot, especially since the COVID-19 lockdown. She does not consider her use of media to be problematic. Staying up late at night, EN often finds herself “doom scrolling” or watching content over streaming platforms. Her parents express concerns about a potential “addiction”. Whenever EN disregards screen time rules that her parents set, they take away her phone, which causes them a great deal of emotional distress. She has subsequently tried to break open the safe where her phone was kept, which surprised both her and her parents.

In line with EN’s presentation,^1^ functional issues relating to youths’ use of popular media technologies (e.g., internet, gaming, and social media) have emerged in various shapes and forms as it has evolved over time (1, 2). These issues often converge towards the dysregulated use of contemporary media (e.g., social media, streaming platforms, gaming), but can span a wide range of media.

Early perspectives characterized this matter in ways analogous to substance use (e.g., tolerance, withdrawal, and wanting/craving) (3), with later perspectives recognizing the importance of core *bio-psychological pathways* (e.g., executive functioning limitations, reward conditioning, and habituation of media-centric coping), *situational factors* (e.g., media availability/accessibility and situational stressors), and *social interaction dynamics* to more fully account for how and why select youth may experience media-related problems (4, 5). While the Interaction of Person–Affect–Cognition–Execution (I-PACE) model (4, 6) outlined key bio-psychological and situational factors (e.g., media availability/accessibility and situational stressors) intermediating problematic use, it largely ignored core social interaction dynamics underpinning problematic use as outlined by the Interaction Theory of Childhood Problematic Media Use (IT-CPU; e.g., oppositional caregiver–child interaction dynamics) (5). In contrast, while the IT-CPU sought to contextualize key maintaining pathways of problematic use, it did not seek to formally integrate core bio-psychological pathways maintaining problematic use as outlined by I-PACE. As a result, established theoretical perspectives contextualizing the etiology and prognosis of problematic media use (PMU) have yet to be formally integrated in ways that capitalize on their primary contributions to this field of study; there exists a need for consolidating theory in the study of media-related impairments among youth (7). Abstracting across and integrating constructs proposed by established frameworks, for example, may provide an improved basis to more inclusively document and chart a patient’s prognosis via the observation of potential/known determinants of PMU at clinical presentation.

Working towards the establishment of a flexible, meta-theoretic framework that more holistically accounts for core *bio-psycho-social and situational dynamics* underlying the experience of PMU would also help to complement existing, comprehensively described theoretical accounts seeking to advance understanding on this topic (4, 5)—particularly by further supporting their application and utility in practice. One way to bolster such efforts could emerge by more formally integrating such frameworks, among others (e.g., process-centric models of emotion regulation) (8), to more comprehensively encapsulate how core maintaining factors may unfold in a lived context to catalyze psychosocial impairments among youth exhibiting dysregulated media use behaviors (e.g., children, teens, and young adults accessing clinical care for media-related problems).

## Purpose and aims

The present study conducted a mixed-methods analysis of data from 205 clinical charts from patient onboarding sessions (ages 9 to 24) referred to a specialty clinic in the US focused on treatment of media use disorders (i.e., Boston Children's Clinic for Interactive Media and Internet Disorders) to advance an integrated, meta-theoretical framework of PMU. To do so, we leveraged generative artificial intelligence (AI) (i.e., GPT-4o) to systematically extract relevant patient information for subsequent qualitative review, a data verification pipeline to validate extracted excerpts from patient charts, content analysis to quantify frequencies in functional impairment across patients, and grounded theory as a basis to thematically analyze patient data/extracted excerpts for the purpose of theory building. While underemployed methodologically (9), medical chart reviews provided the unique opportunity to obtain a vivid picture of the lives of young people struggling with their media use, affording the opportunity to derive rich qualitative insights (10). Results suggested that consideration of a given person (P—Person) in their lived context (C—Context) is necessitated to contextualize the processes (P—Process) underpinning PMU, their likely outcomes (O—Outcomes), and capacity to compound psychosocial impairment over time (T—Time). To help structure such inquiries, we proposed the Person–Context–Process–Outcome–Time (PC-POT) model of PMU to better connect and supplement existing theoretical frameworks.

### A need to build from extant diagnostic criteria

**Internet gaming disorder**. Considering that clinical criteria for media use problems are already defined, they can help shape our understanding of the core processes underpinning problematic use. For instance, internet gaming disorder (IGD) proposed by the *Diagnostic and Statistical Manual of Mental Disorders* (*DSM-5*) (11) defined symptoms that span both *bio-psychological* (e.g., preoccupation, loss of control, and media-centric coping) and *social* domains (e.g., deception and interpersonal/vocational impairment). Select criteria (e.g., deception and media-centric coping), however, can clearly vary in their relevance on a person-by-person basis (for a discussion, see (12)). Other IGD criteria maintain less bearing on characterizing problematic use due to their limited support (e.g., tolerance and withdrawal) (13, 14).

Gaming disorder. The need to further standardize a more universal set of diagnostic benchmarks informed the later introduction of gaming disorder (GD) proposed by the World Health Organization’s (WHO) *International Classification of Disease* (*ICD-11*) (14, 15). GD, as a refinement of IGD, centered on *impaired control over gaming activities*, *increased priority given towards gaming over non-gaming activities*, and *persistent use despite the experience of negative consequences* as diagnostic criteria (14). Like IGD, impairment for at least 12 months or more is necessitated to warrant a diagnosis of GD by WHO’s guidelines (11, 14, 15).

**Limitations of a diagnostic approach**. While a recent Delphi study of experts supports GD’s improved diagnostic utility over IGD (13), a diagnostic approach to understanding PMU maintains some inherent limitations. First, existing diagnostic criteria (e.g., IGD) remain limited in their scope (e.g., 1, 16). As in EN’s case, media-related impairments can span multiple types of media, not just gaming; meta-analytic results demonstrate that problematic use of social media, for instance, is positively associated with IGD over time (*r* = 0.24) (35). Second, GD/IGD criteria rely on impairment persisting for 12 months or more (14), which remains largely antithetical to the early intervention of PMU to the potential detriment of treatment efficacy (16). Third, even if a clinical cutoff is not met for GD/IGD, assessment around appreciable forms of media-dependent harm is warranted (e.g., see hazardous gaming) (14, 15) and may even fall outside the criteria’s scope of assessment (e.g., caregiver perceptions/beliefs about their child’s media use) (5). Last, IGD/GD hinge on a subjective judgment made around experienced harm attributed to media use (11, 12, 14, 15). Harm judgments can vary across persons and cultures (17, 18), however, suggesting that the primary criteria for a GD/IGD diagnosis is (to one degree or another) more or less subjective in nature. Thus, GD/IGD as diagnostic criteria should not function as the sole basis for investigating or treating PMU.

Current IGD/GD formulations also speak to bio-psycho-social dynamics that can naturally coincide with psychosocial impairment for some vulnerable young people over the course of development, necessitating caution around pathologizing media use without consideration of underlying individual susceptibility factors (15, 16). The comorbidity between autism spectrum disorder (ASD) and IGD/GD, for example, is at times attributed to unique social needs (e.g., challenges interpreting social cues), narrowed special interests (e.g., revolving around media activities), and a preference for routine activities typified by ASD (19, 20). In contrast, an increased preference for sensation seeking, heightened rate of externalizing behaviors, and diminished inhibitory control typified by attention-deficit hyperactivity disorder (ADHD) is often used to explain its comorbidity with GD/IGD (20, 21). Both ASD and ADHD associate with cognitive impairments (19–21), with deficits in executive functioning generally predisposing engagement in persistent, non-adaptive modes of behavior (22). These and other susceptibility factors (e.g., increased sensation seeking, hypersensitivity to social feedback/rewards, executive functioning limitations, preference for and habitual engagement with media, and psychosocial problems), however, also maintain parallels within the scope of normative development (see (23, 24); for a review of adolescent development and media use, see (25)).

In all, the culmination of proposed diagnostic criteria (e.g., dysregulated media use and psychosocial problems) may (at times) represent a by-product of commonly experienced *bio-psycho-social* dynamics (e.g., reliance on media use for emotion regulation, dysfunctional parent–child media use dynamics, and executive functioning limitations associated with normative development) (4, 5), prompting debate around its characterization as a single or uniform pathological condition (16). Taking a *hardline confirmatory stance* towards pathologizing generic forms of media use would also neglect the fact that media use is situationally dynamic, personalized in its scope, and evolving in its contemporary design over time (5, 26, 27). Thus, *gaming* (like other forms of media use) *cannot universally confer* dysfunction as to support such a disordered view of IGD/GD *when considered on its own as a monolith*, unlike substance use (10). Rather, dysfunction resulting from media use emerges as a complex interplay between a given person’s individual susceptibilities (e.g., level of development and disposition), their offline environment (e.g., parent–child dynamics), and the substance of their personalized media environment (4, 5, 26–29). A holistic account of PMU, therefore, warrants due consideration of the capacity of a given person (P—Person) and their media use in context (C—Context).

### A need to center process over classification

Taking a person- and process-centered approach has the potential to help inform on the core factors underpinning PMU in line with the Research Domain Criteria (RDoC) framework proposed by the National Institute of Mental Health (30). For instance, the process underwriting the motivational shift (i.e., incentive sensitization) towards a compulsive pattern of behavior (e.g., gaming) is not inherently pathological, but the predisposition to give into temptation resulting from heightened sensations of wanting over one’s day-to-day life can lead to pathology and dysfunction (31). In line with the RDoC framework (32), research efforts have shifted towards a more process-centric understanding of PMU (e.g., 4, 5).

### Centering process-centric frameworks of PMU

Two recent theoretical frameworks seeking to explain the etiology of problematic use partially account for the development and maintenance of dysregulated media use dynamics from a *bio-psycho and/or social* perspective. To our knowledge, however, they have yet to be formally integrated.

I-PACE. Perhaps the most comprehensive account of PMU is the I-PACE model (4, 6). It is a neuro-biological account that outlined how bio-psychological factors (e.g., impaired inhibitory control, incentive sensitization, habituation) interplay with situation-specific factors (e.g., negative affective states, incentive salience, and media accessibility/availability) to promote problematic use via a self-reinforcing set of person–situation dynamics. According to I-PACE, select links in affect–cognition strengthen (e.g., negative affect and media-centric coping strategies) and stimuli-specific inhibitory control weakens in ways that facilitate more automatic/habitual media use behaviors over time, with media-specific cues aiding to catalyze this process (e.g., intermittent rewards). General deficits in inhibitory control can also promote the stabilization or intensification of problematic use, which is primarily driven by reward conditioning (e.g., incentive sensitization towards specific media cues). Media-centric coping, as a way to manage negative affect resulting from situational stressors, also helps to solidify the stabilization/intensification of problematic use via habituating select emotion regulation strategies over time (e.g., avoidance). *Loss*, *frustrated non-reward*, *anxiety*, *fear*, and *boredom* all serve as potential *negative affective states* underpinning “compulsive” behavior (33, 34).

In line with I-PACE, self-control, time spent gaming, negative affective states, and situational stressors (e.g., abuse by family and loneliness) associated with IGD over time per a recent meta-analysis of longitudinal studies (35). Time spent gaming served as the strongest predictor of IGD (*r* = 0.33), with self-control serving as the strongest protective factor (*r* = −0.27); negative affective states (e.g., loneliness and anxiety) and situational stressors (e.g., early life adversity) also operated as risk factors with IGD over time; loneliness exhibited the second largest effect size with IGD (*r* = 0.29). These results would suggest that incentive sensitization, time spent using media, impaired inhibitory control, and the habituation of media use for emotion regulation may play a central role in underpinning PMU for a given young person (e.g., EN).

IT-CPU. Moving beyond I-PACE, the IT-CPU sought to center social interaction dynamics and ecological theory [e.g., Process–Person–Context–Time (PPCT) model (36);] to better account for problematic use over development (5). Importantly, in a developing person’s most immediate environment (i.e., microsystem), there exists a set of *proximal processes*, or reciprocal interactions between a given person and their immediate environment, functioning as the primary drivers of development/psychosocial functioning (36). The IT-CPU outlined several proximal processes associated with the development and maintenance of problematic use during development (e.g., oppositional caregiver–child interaction dynamics) that can operate via distinct psychological mechanisms, like conditioning (e.g., psychological reinforcement), social learning (e.g., modeling), and social influence (e.g., perceived norms) (5).

Social interaction dynamics can operate as a core determinant of PMU, which centers on caregiver–child interactions over early development (5). Meta-analytical results demonstrated that parental withdrawal (e.g., lack of interest in the child’s activities) (*r* = 0.28) and overinvolvement (e.g., excessive control) in a child’s activities (*r* = 0.186) associated with increases in IGD (37). A meta-analysis of longitudinal studies of IGD also exhibited similar results; positive parent–child relationships (*r* = −0.15) and parental supervision (*r* = −0.09) also served as protective factors (35). In all, these findings suggested that parental/caregiver involvement is necessitated for effective media use during childhood and adolescence, but within the bounds of developmentally appropriate, autonomy supportive parenting (35, 37). Too much or a lack of parental/caregiver involvement may, thus, partially underpin the experience of PMU for some youth.

### Situating PMU in a lived context: person (P—Person) in context (C—Context)

The need to more formally interlink core aspects of the IT-CPU and I-PACE supports additional open-ended, qualitative investigation of PMU—specifically, to advance the development of a more fully integrated and situationally informed *bio-psycho-social* framework. Despite idiosyncratic differences across people/patients and the familial, social, and physical contexts they remain embedded within, *a transferable set of potentially hazardous media use dynamics* may emerge to help account for why and when media use may pose appreciable harm (see 15). One hazardous media use dynamic centered in proposed diagnostic criteria included the displacement of non-media-related activities with preferred media in ways that are associated with psychosocial dysfunction (e.g., staying up into the early morning playing video games paired with daytime fatigue) (14, 15). The distillation and identification of themes around the underlying determinants of problematic use, when extracted from real-world accounts of youth reporting media use problems, could help better pinpoint the factors fundamentally underpinning PMU as an experiential cycle of media-related dysfunction in line with RDoC’s process-centric emphasis (30). Therefore, we propose the following research question:

RQ1: What types of hazardous media use dynamics exhibit themselves among patients in the US accessing *clinical treatment* for PMU?

## Methods

### Sample

This qualitative chart review study was deemed exempt from human subjects oversight after review by the Boston Children’s Hospital (BCH) Institutional Review Board (IRB). Medical charts—from each patient’s initial evaluation meeting with the clinician—were sourced from a specialty clinic focused on addressing media use problems and served as the basis for our mixed-methods analysis. The first clinical session focused on gathering background information about the patient, in addition to documenting potential media-related impairments. Just over half of sessions were conducted virtually as a telehealth visit (50.24%). Patient visits spanned seven clinicians in total, with Clinician 1 (62.44%) and Clinician 2 (29.76%) conducting most intakes.

Patient onboarding sessions, totaling 205 patient charts (86.34% male, 12.68% female, 0.98% other), were available for review (conducted from July 2017 to November 2021). Patients ranged in age from 9 to 24 years (*M* = 14.79, *SD* = 2.408). More than half of the patients (56.59%) identified themselves as White, 6.83% Hispanic or Latino, 3.90% Black or African American, 2.93% Asian, and 13.17% other; under one-quarter of patients (23.41%) did not provide any race-based demographic information. Diagnosed mental disorders among patients included ADHD (61.95%), anxiety (45.85%), mood disorders (30.73%), ASD (14.15%), oppositional defiant disorder (ODD) (13.17%), and obsessive–compulsive disorder (OCD) (5.85%). Of the disorders listed, most patients had at least one diagnosis (78.54%) and over half had two or more (55.12%).

### Plan of analysis

The present chart review spanned several research phases outlined by Gearing and colleagues (9), including conception (i.e., clinical scan and research formulation), literature review, development (i.e., defining variables), and theme extraction in line with our primary research question. To thematically analyze patient charts, we employed an iterative, mixed-methods approach to leverage the strengths of our cross-disciplinary research team (i.e., spanning personal with an expertise in cognitive psychology, neuroscience, media and technology, clinical pediatric care, and social work) and the theory building elements afforded by grounded theory (38). The project took on two phases to address RQ1. Patients’ medical charts were anonymized by removing/replacing personal identifiers (e.g., formal names of person/institutions), in addition to the results of specific medical tests, prior to data analysis.

**Figure 1:**
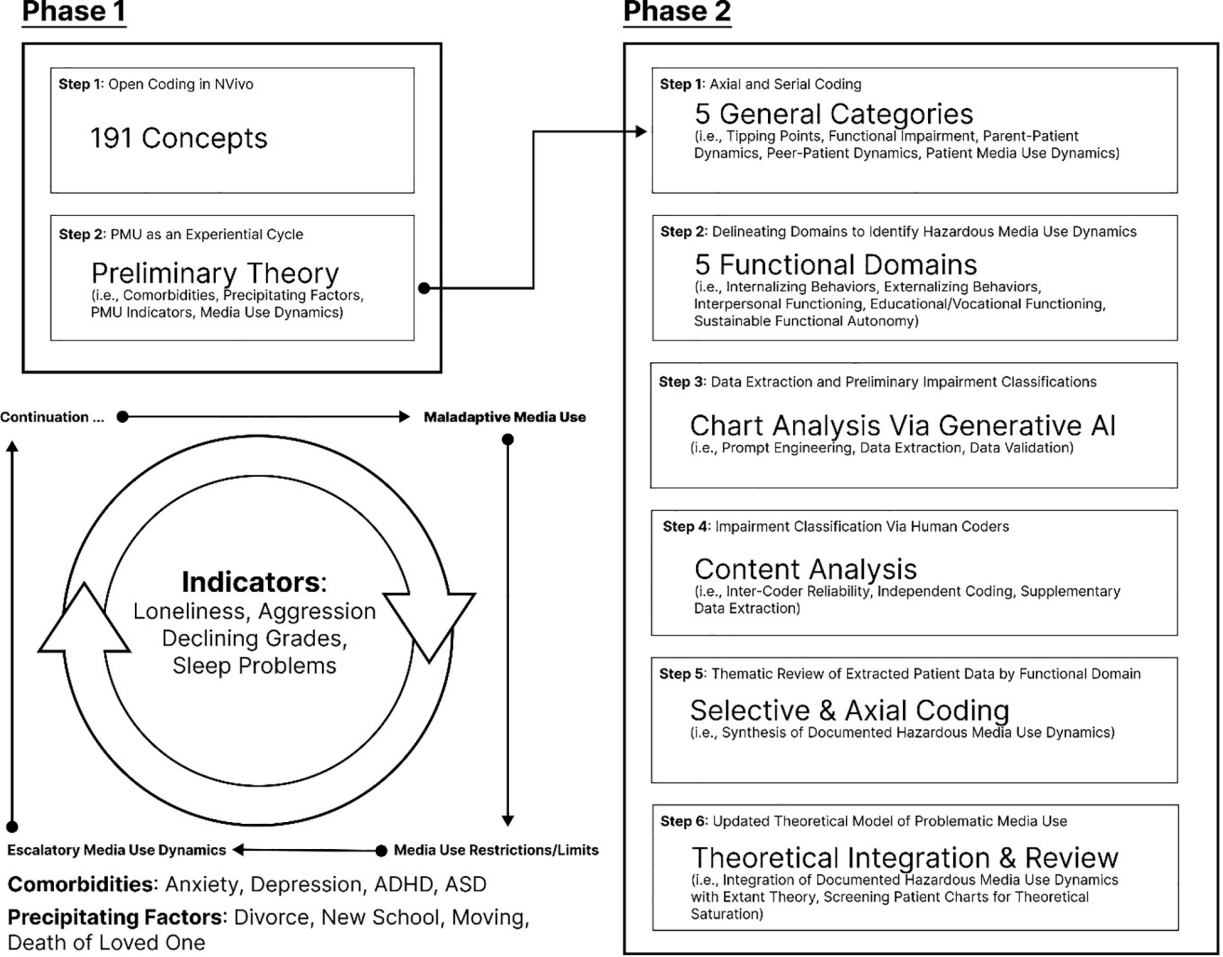
Overview of research phases. The research process took on two separate phases (i.e., Phase 1 and Phase 2); the second built on the first.

Phase 1 included *open coding* in Nvivo (e.g., inductive deriving concepts based on patient data), in addition to an iterative process of *axial* and *selective coding* (e.g., exploring/defining concept relations and concept abstraction); a subset of the research team (two authors and two research assistants) identified a set of constructs and concept relations relevant to their knowledge of PMU using a grounded theory approach (38). This yielded a preliminary model of PMU as an experiential cycle (see **Figure 1**). Reliability was computed pairwise between coders progressively during the initial training and coding process. Simple agreement (averaged 72.60% across the four coders) across a subsample (approximately 20%) of entries exhibited a high degree of overlap between the coders. The final codebook contained 191 coding categories.^2^ At a high level, preliminary codes included select *transition-linked turning points* (e.g., new device, COVID-19 lockdown, moving, and death of a loved one), *patients’ media activities* (e.g., characterizations of their media use as excessive in amount or risky/inappropriate by type and sneaky media use), *parent–child dynamics* (e.g., parent–child conflict around media and media rules/restrictions), *child-peer dynamics* (e.g., using digital media to engage socially), and different forms of *functional impairment* (e.g., sleep problems, poor diet/hygiene, aggressive behaviors, social withdraw, and declining grades).

Phase 2 repeated aspects of Phase 1, but included a refined process of *open* and *axial*, and *selective coding* using a combination of human input and generative AI. Specifically, from the 191 initial codes, a separate segment of the research team unrelated to Phase 1 derived a final set of functional domains spanning five categories to serve as a basis for identifying hazardous media use dynamics: (1) internalizing behaviors, (2) externalizing behaviors, (3) interpersonal functioning, (4) educational/vocational functioning, and (5) sustainable functional autonomy. Definitions of each domain and how they were derived are included in our **Supplementary Materials**.

Definitions of each domain were provided to ChatGPT as a means to (a) systematically classify impairment by domain per patient chart and (b) extract excerpts from patient’s medical charts encompassing hazardous media use dynamics for our final thematic review (RQ1). Doing so provided a way of facilitating a *more objective, scalable, and reproducible process of data extraction* from patient charts based on a defined set of social knowledge (i.e., AI model and shared prompt). We used a BCH secured ChatGPT-4o API to analyze patient data; the security measures ensured that the data remained within the hospital’s protected data infrastructure, preventing it from being stored or analyzed by a third party (e.g., OpenAI), thereby safeguarding patient privacy and security. The first prompt outputted one binary classification for each domain (1 = present, 0 = absent), along with excerpts it used to support each positive classification. The prompt included a set of media-dependent impairment examples, but did not include instructions to source media-specific excerpts. The second prompt tasked the AI to extract supplementary excerpts for each functional impairment classified as present by the first prompt. The second prompt excluded any media-dependent impairment examples, but included instructions to source documented associations linking media use with impairments by domain. Excerpts were validated using an automated coding pipeline to ensure those reviewed to address RQ1 were not fabricated. All functional impairment classifications were reviewed by human coders. Please refer to our **Supplementary Materials** for additional details on our prompt engineering and output validation steps.

Theoretical integration. Additional concepts were sourced to theoretically integrate evidenced media use hazards observed across excepts, in line with I-PACE and the IT-CPU, using a grounded theory approach. How a person views and uses media represent the primary drivers of PMU across both accounts (4, 5), along with how others in their immediate environment (e.g., parents, teachers as caregivers) perceive and respond to their media use (5). Changes in perception, beliefs, or behavior around the given person’s media use *in situ*, therefore, always functionally complete a segment in a broader chain of events underpinning someone’s experience of PMU. The recurrence of select media-dependent cognitions/behaviors may then compound PMU over time (4, 5).

One way to universally contextualize divergent life trajectories and emergent psychosocial impairments associated with PMU includes the concept of *turning points*—events or circumstances that accentuate or alter the internal or external state of an individual in ways that may catalyze psychopathology (39). While life is riddled with various turning points, some turning points directly result from specific transitions, known as *transition-linked turning points* (39, 40). As turning points can operate as by-products of an individual’s behavior and even result from outside influences (e.g., social circumstance) (39), the application of transition-linked turning points as a concept to help characterize specific “transitions” associated with change in a young person’s conception or use of media specifically is possible, irrespective of the bio-psycho-social factors at play. Thus, we leveraged the concept to help contextualize extracted excerpts.

**Figure 2:**
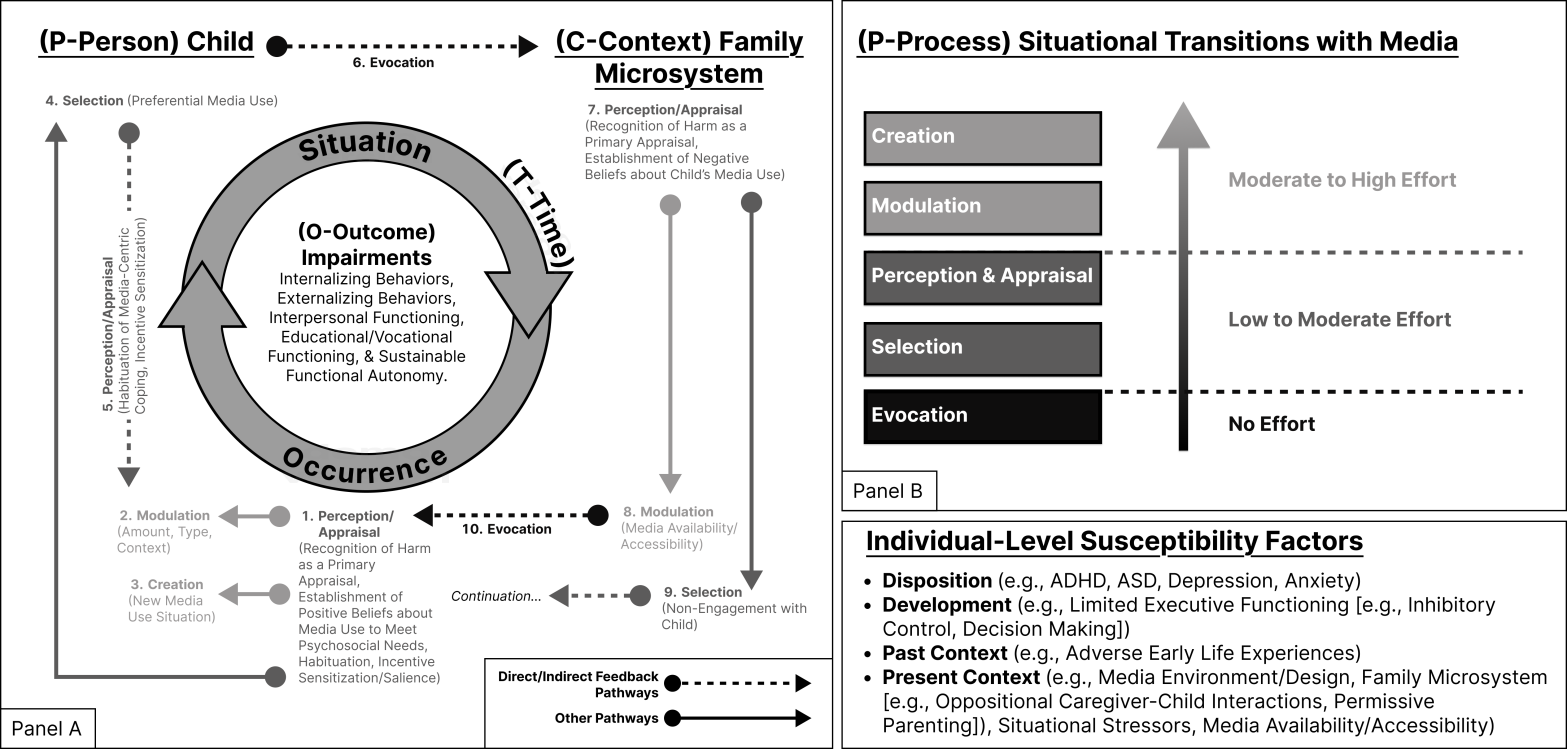
Person–Context–Process–Outcome–Time (PC-POT) model of problematic media use. Changes in problematic media use (PMU) dynamics emerge due to the occurrence of select situational transitions with media (P—Process), which catalyze situational change over time in ways that can lead to or compound psychosocial impairment (O—Outcome). Given a person (P—Person) in context (C—Context) (e.g., media environment and family microsystem), it is possible to map out the pathways (P—Process) promoting the onset, maintenance, or exacerbation of media-related impairment (O—Outcomes) over time (T—Time) (i.e., PC-POT). Within the context of PC-POT, each situation transition encompasses a perceptual/behavioral change related to media with the potential to alter or maintain PMU dynamics, provided their occurrence, stabilization of new dynamics, and recurrence. *Evocation* represents a circumstance wherein media use elicits an incidental response (from another) in a given situation; *perception/appraisal* represents a change in one’s conception of media use activities (e.g., habituation of media centric coping, incentive sensitization to media-related cues, recognition of harm as a primary cognitive appraisal, and change in decision-making around media use as a secondary cognitive appraisal); *selection* represents an approach or avoidance tendency towards one situation over another as to facilitate media use; *modulation* denotes actively altering an existing media use situation (e.g., amount/intensity, context, availability/accessibility, and type); and *creation* includes the pro-active creation of a new media use situation, not just the alteration of an existing media use situation. Situation transitions vary in their degree of effort (41, 42), biasing engagement towards lower effort situational transitions with media in ways that increase the rigidity of PMU as an experiential cycle of media-related dysfunction.

At the level of an individual, each type of transition could effectively map onto one of several *situational navigation mechanisms*—which mirror and extend beyond concepts employed by theories of emotion regulation (8)—to demarcate how individuals may intentionally or unintentionally alter their situational circumstances (41, 42): *evocation*, *selection*, *construal*, *modulation*, and *creation*. *Evocation* represents the incidental elicitation of a response (from another) in a given situation. *Selection* encompasses conditioned approach and avoidance tendencies towards one situation over another. *Construal* (which we subsequently just refer to as *perception/appraisal* in line with emotion regulation frameworks) (8) encompasses a change in someone’s understanding, attitudes, and/or beliefs around a particular situation. *Modulation* denotes the active alteration of a pre-existing or ongoing situation. Lastly, *creation* constitutes circumstances where someone proactively constructs an entirely new situation. Each concept effectively helps to interlink the primary contributions of I-PACE and IT-CPU by tethering together *bio-psycho*, *situational*, and *social* dynamics determinants of PMU using *media-related transitions as a linking factor*. A description of each situational transition with media in the context of PC-POT is provided in **Figure 2** (see notes).

**Figure 3:**
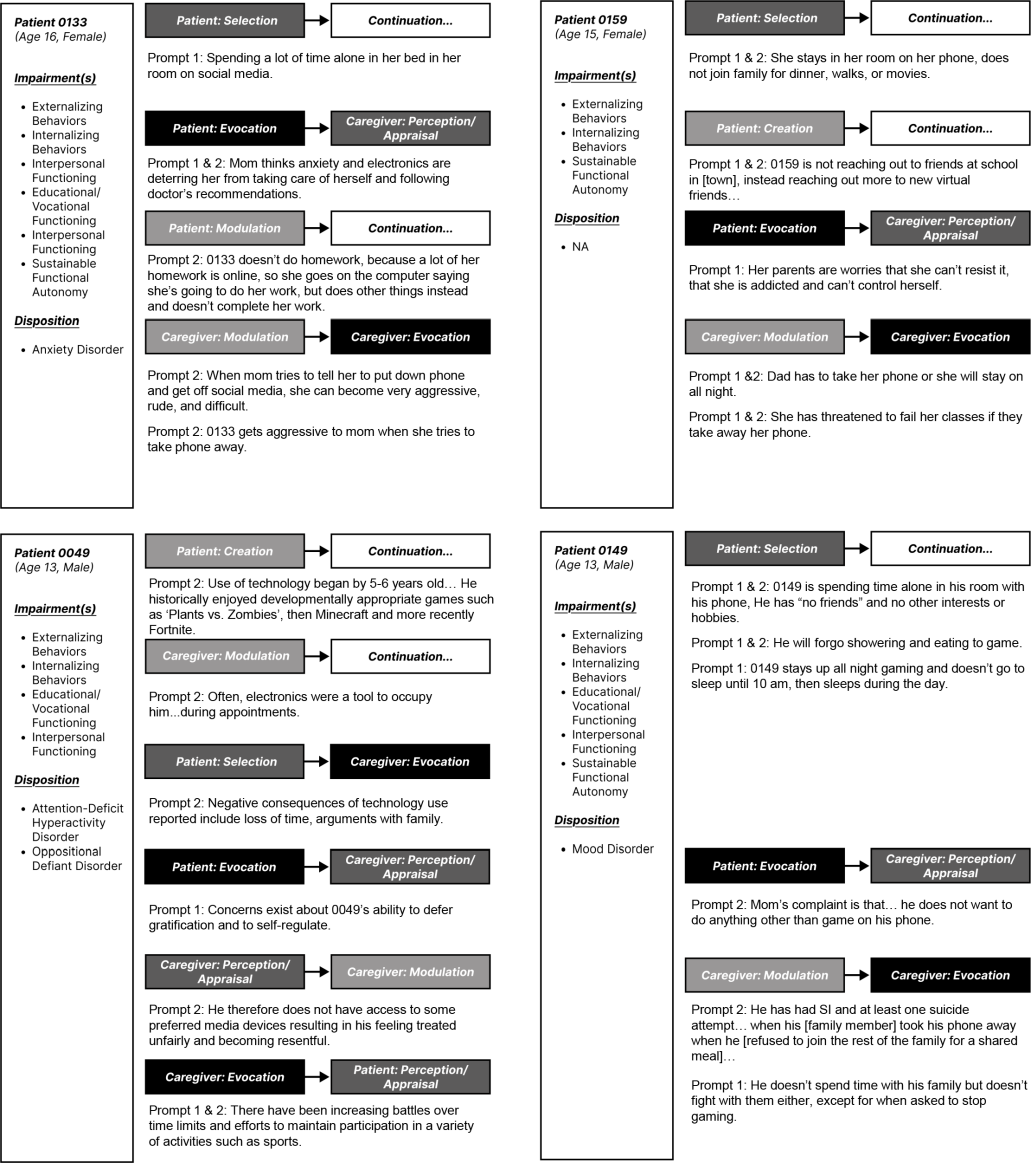
Situational transitions with media: Example transitions and linkages. *Notes.* Some excerpts denoted linkages between select situational transitions with media. Core pathways are included above, except for selection (as avoidance) on part of a caregiver. Patient diagnoses and impairment classification(s) are listed below the anonymized patient identification number. For brevity, only select excerpts of those extracted are illustrated.

Since functional impairment serves as the focal component of patient medical charts, classification percentages for each functional domain by patient chart are presented in aggregate. Because background information was oftentimes limited to parental reports and differed in its veracity across patient charts, a high-level thematic overview of documented *hazardous media use dynamics* was provided to directly address RQ1. Hazardous media use dynamics were identified through review of patient charts with the goal of identifying patterns of sequential events leading to substantial behavioral disruption at clinical consultation (see **Figure 3**). In this way, the approach is similar to chain analysis in dialectical behavior therapy (43). Extracted media-related excerpts functioned as the basis for deriving an updated working model of PMU during Phase 2 based on the preliminary model created during Phase 1. A manual review of the same subsample of patient charts used for intercoder reliability functioned as a basis for establishing theoretical saturation, irrespective of the AI output. To reduce the inclusion of peripheral patient-related information, select details across extracted excerpts were either omitted (e.g., "...") or paraphrased (e.g., "[their state]") during the review processes. Edits did not change the substance of the information provided.

## Results

### Externalizing behaviors and modulation

Instances of externalizing behaviors among patients at presentations were common (AI, 75.12%, *n* = 154; Human Coder, 82.93%, *n* = 170). Such behaviors range from oppositional behavior (e.g., not handing over devices when asked, sneaking media use when restricted, deception about media use, and bypassing screen time management software), theft/deviancy (e.g., stealing money to purchase digital goods/services), verbal (e.g., fighting with parents and threats of self-harm) and physical forms of aggression (e.g., damaging property, injuring others, and self-directed violence), harassment (e.g., sexual harassment), substance use, and impulsivity. Some characteristic examples included the following:

*Prompt 2 - ID:0045 - 0045 lies lies about computer time, has stolen money from credit cards to buy games.*
*Prompt 2 - ID:0009 - He was recently aggressive with Mom because she took away xBox. He knocked Mom down and tried to break her door to get it back. He has punched holes in the wall.*
*Prompt 2 - ID:0096 - 0096 has smashed and broken things when parents turned off the internet. This sparked parents to call the police.*
*Prompt 2 - ID:0149 - He has had SI and at least one suicide attempt… when his [family member] took his phone away when he [refused to join the rest of the family for a shared meal]…*

Most of the time, oppositional behavior and conflict (e.g., refusing to hand over devices and not stopping use when asked/demanded) reactively stemmed from the proposal or implementation of media use restrictions (i.e., *modulation*) by a caregiver (e.g., parent and school staff). Abrupt attempts to cuff off access (at times) prompted circumstances warranting emergency care or assistance.

### Internalizing behaviors and selection

Instances of internalizing behaviors among patients were common (AI, 87.32%, *n* = 179; Human Coder, 75.12%, *n* = 154). This included everything from anxiety/depression symptoms, impaired emotion regulation, and patients socially isolating themselves. Some characteristic examples included the following:

*Prompts 1 and 2 - ID:0009 - He is anxious and depressed and has PTSD from having witnessed and possibly experienced domestic violence.*
*Prompt 1 - ID:184 - One on one, 0022 acknowledges that she often feels sad.*
*Prompt 2 - ID:0008 - Gets to school and “can’t find the courage to go in … paralyzed with anxiety”.*
*Prompts 1 and 2 - ID:0022 - Her mom’s concern is that 0022’s recent Instagram posts have turned sadder, darker, and more desolate, culminating in an image of a gloved hand with pointed finger hovering over a doorbell-type button with the caption, “To die, press here.”*
*Prompts 1 and 2 - ID:0074 - He states that it has been identified that he feels less nervous when playing video games and finds that it is a distraction from his fears.*
*Prompts 1 and 2 - ID:0102 - Parents stated that they believe that the anxiety gets in the way of him being able to do difficult things and that patient ends up avoiding and numbing with interactive media.*

*Selection* of media use activities as a tool to manage negative affective states (e.g., loneliness, anxiety, and fear) was common.

### Interpersonal functioning and selection

Interpersonal functioning impairments across patient charts were common (AI, 88.29%, *n* = 181; Human Coder, 86.83%, *n* = 178). This included everything from declining to reciprocate a willingness to engage socially with known others (e.g., siblings and parents), exhibiting difficulties navigating in-person interactions (e.g., interpreting non-verbals, conversing, and social anxiety), and not wanting to have certain types of interpersonal relationships at all (e.g., friends). Whereas conflict around media use also emerged across charts as a common point of interpersonal tension (e.g., accessing “inappropriate” content and ignoring others while using devices), patients often exhibited a lack of effective conflict resolution strategies; they often expressed a preferential desire (i.e., *selection*) to engage with media in place of many (if not all) social activities offline. Forms of socialization across patients, if reported, were often convened over digital spaces (e.g., social media and gaming). Some characteristic examples included the following:

*Prompts 1 and 2 - ID:0016 - He says that he has no friends and no desire to make friends.*
*Prompt 2 - ID:0024 - Decrease in live social contact (less time with mom, family, and cousins who he is close with, does not have many friends, on the autism spectrum, does not enjoy hiking or going to beach anymore).*
*Prompt 1 - ID:0159 - She is very shy face-to-face and has difficulty talking, so she avoids real people. But she is very outgoing online—mom says it is as if she has 2 personalities.*
*Prompt 2 - ID:0073 - Parents state that 0073 has had issues with “reading social cues” before, that she struggled to establish a friend group growing up. She tends to primarily play with her younger sister.*
*Prompt 2 - ID:0162 - But underlying anger toward mom and unwillingness to trust her enough to repair relationship appear to be driving gaming behaviors as distraction and self-soothing.*

Displacement of offline social activities, due to preferential *selection* of media over non-media-related activities, was commonplace across patient charts.

### Educational/vocational functioning and selection

Educational/vocational functioning impairments across patient charts were common (AI, 84.88%, *n* = 174; Human Coder, 77.56%, *n* = 159). This included everything from problems around completing required tasks (e.g., inattention, poor time management) and non-engagement/deferral of engagement in academic/extracurricular activities to declining grades/failing a grade level and even absenteeism/withdrawing from school. Some patients even demonstrated a lack of interest in school as a means for attainment and employment issues. Some characteristic examples included the following:

*Prompt 1 - ID:0028 - Per mom, he will go on YouTube during his 5-min breaks, become consumed and then skip the next class.*
*Prompts 1 and 2 - ID:0011 - He feels that school “is not as important” and “I want to be a professional gamer when I grow up”.*
*Prompt 2 - ID:0030 - 0030 was an honor roll student in honors classes, and is now failing 4 classes.*
*Prompts 1 and 2 - ID:0047 - 0047 is very bright and did well in school until she started gaming, but this year, she was failing, and withdrew from school.*

A lack of engagement in educational/vocational tasks, due to preferential *selection* of media over non-media-related activities, was commonplace across patient charts.

### Sustainable functional autonomy and selection

Instances of sustainable functional autonomy (SFA) impairments at patient presentation were common (AI, 84.39%, *n* = 173; Human Coder, 77.56%, *n* = 159). SFA spanned everything from instances of under-medication (e.g., under-medicated ADHD symptoms) and disturbed sleep to self-care issues (e.g., poor diet, lack of hygiene, and lack of exercise) and neglect of daily life tasks (e.g., chores). Some patients also demonstrated a lack of motivation to do anything at all, often with the exception of media use. Some characteristic examples included the following:

*Prompts 1 and 2 - ID:0031 - Parents express concerns of 0031 missing out on meals, showering, and physical activities.*
*Prompt 2 - ID:0030 - Mom has changed the passwords on video streaming channels and 0030 is not allowed electronics after 8 p.m. on school days, has been skipping meals, has no physical activity, ...was not showering, and carries electronics to the bathroom with him.*
*Prompt 1 - ID:0037 - He is staying up late or midnight waking up in the night to play. Has difficulty waking up in the morning.*

Displacement of necessitated daily activities, due to preferential *selection* of media over non-media-related activities, was commonplace across patient charts.

### Transition-linked turning points

General transition-linked turning points. Multiple patients reported experiencing various types of transition-linked turning points that appeared associated with later psychosocial problems and their use of media. This included everything from death of a loved one and familial conflict (e.g., divorce/separation) to events impairing in-person social contact (e.g., COVID-19 lockdown) and even moving/changing schools. Academic stress was also common and can be associated with increased academic demands over time. Some characteristic examples included the following:

*Prompt 1 - ID:0116 - Since the pandemic began, 0116 has been mostly staying in his room all day, at times neglecting meals, showering, and will stay up all night.*
*Prompt 1 - ID:0135 - Anxiety - Describe as excessive worry and agitation in particular with “change and transitions”.*
*Prompt 2 - ID:0092 - As she had less access to in-person social contact, she began to rely more upon social media for socializing.*
*Prompts 1 and 2 - ID:0079 - Negative emotional states - Grief/dysphoria related to [sibling's] death [occurred over a year prior].*
*Prompts 1 and 2 - ID:0098 - [Father] stated that it is possible that it could be a way to “escape” or avoid his feelings around the divorce, spending 50/50 time with both parents, and moving to a new school.*
*Prompt 2 - ID:0034 - Negative emotions related to academic demands.*
*Prompt 1 - ID:0068 - About to start new school year with heavier academic load with more required screen time.*
*Prompt 1 - ID:0004 - 0004’s and his siblings’ exposure to parental conflict has led to each of them withdrawing into their rooms, often in bed under their covers using smartphones or tablets to game, social media, or watch videos. They are afraid to talk or even emerge.*

Other types of (transition-linked) turning points more directly linked to the patient’s media use were common, however. Those listed above, however, were often demarcated as a reason for *media use as an emotion regulation tool* (explicitly or implied).

Establishing new media use situations: Creation. The establishment or pursuit of new media activities also seems to partially underpin PMU, particularly by leading to new media use situations. This would include establishing a digital presence (e.g., social media account) or new digital relationships (e.g., online friends), but also getting new devices or onboarding new digital spaces (e.g., platform/game). Some characteristic examples included the following:

*Prompts 1 and 2 - ID:0159 - 0159 is not reaching out to friends at school in [town], instead reaching out more to new virtual friends that do not live in [their state].*
*Prompt 1 - ID:0050 - Per 0050’s parents, they noticed a change after [holiday] at which time 0050 had gotten a new computer as a gift, which he built himself for gaming and ... school.*
*Prompts 1 and 2 - ID:0154 - When he started playing Fortnite, 0154 became increasingly impulsive in his behaviors and aggressive in his language, and when parents tried to get him off the game, he became destructive of TV remotes and other items.*
*Prompt 2 - ID:0088 - History with media use? Connect with people, has new Instagram for.*

Selection as preferential media use and avoidance. Many examples of *selection* as preferential media use emerged across patient charts in ways linked to the patient’s psychosocial functioning (see excerpts above). However, *selection* as avoidance by caregivers/others also appears to partially underpin a child’s experience of PMU—particularly by enabling further engagement with media. While more apparent among children themselves due to *selection* of media over non-media activities, some excerpts supported *selection* as preferential form of disengagement with or avoidance from the patients themselves. Media use also coincided with reports of ongoing conflict and interpersonal tensions—arguably factors discouraging the initiation of social engagement by others outside the patient’s use of media. Some characteristic examples included the following:

*Prompt 1 - ID:0043 - He makes others socially uncomfortable that they avoid him.*
*Prompt 1 - ID:0159 - She has become easily angered since this past summer. She screams and is rude to her parent, mean to her brother (2 years younger), and calls mom names.*
*Prompt 2 - ID:0140 - He does acknowledge less interactions with parents due to his screen-related activities.*
*Prompts 1 and 2 - ID:0198 - There has been a lot of tension and fighting with family, but gets along fine with friends when he is allowed to game online with them.*

Changes in perception/appraisal of media activities. Changes in *perception/appraisal* of media use activities can underpin the experience of PMU. Reports of losing control/preoccupation (presumably due to habituation of media activities, incentive sensitization, and media-centric coping) were well documented across patient charts. Some excerpts also encompassed patient *perceptions/appraisals* of media use restrictions as unfair/overly restrictive (i.e., harmful/damaging), vigilance/concerns around online harassment (e.g., cyberbullying), and/or concerns for the wellbeing of others (e.g., friend). Some characteristic examples included the following:

*Prompt 2 - ID:0025 - They worry that some of his use is “too much” and seems to have become more “obsessive.” They also have concerns about some of the specific content he views.*
*Prompt 2 - ID:0178 - Mom was worried about 0178 playing Fortnite and was worried it would distract him from his grades.*
*Prompt 2 - ID:0010 - 0010 viewed these restrictions on her phone as very damaging to her social life and her ability to socialize with her peers.*
*Prompt 1 - ID:0140 - He acknowledges feeling happier and more positive when playing games and he denies much in the way of any negative consequences of games.*
*Prompts 1 and 2 - ID:0021 - In addition, he stated that when he is having a bad day, he wants to play games to re-regulate; however, he notices that he is more vulnerable to “getting mad easily”.*
*Prompt 2 - ID:0083 - She is taking on her friend’s troubles as they are her own. Prompt 2 - ID:0083 - “always to herself”, staying close to device so her friend can reach her at all times.*
*Prompts 1 and 2 - ID:0297 - When he attempts to stop the behavior, he experiences good amounts of preoccupation and has a hard time refraining himself from thoughts about accessing the material.*
*Prompt 1 - ID:0149 - 0149 says he is going to make a lot of money gaming and that is the only thing important to him.*
*Prompts 1 and 2 - ID:0192 - 0192 presents as anxious, insecure, and sad ...series on incidents online that were precipitated by a video from a [social event] last fall.*

Altogether, this outlines a common juxtaposition in *perception/appraisal* across caregivers and children themselves. Over time, children habituate to perceive media use activities as increasingly positive/rewarding (e.g., means for attainment and way to cope), whereas caregivers (eventually) came to regard their child’s use of media as a primary cause of harm in many circumstances. Engagement with media also remained, at times, contextualized by a concern for others or resulted from vigilance/coping around ongoing events (e.g., cyberbullying), varying across participants by circumstance.

Modulation of media accessibility/availability and evocation. Lastly, *modulation* of the amount/intensity of media use, context of use (e.g., using media at night to avoid detection), or type of media (e.g., switching devices when the internet is turned off or device is taken) is often associated with parental concerns (i.e., *perception/appraisal* as harm judgements), caregiver’s *modulation* of media use activities (i.e., media accessibility/availability), and incidental responses (i.e., *evocation*) from patients themselves due to experienced distress (see **Figure 2**, Caregiver and Child pathways). Some characteristic examples included the following:

*Prompt 2 - ID:0043 - When he did not have a device, he stole family devices to access porn.*
*Prompt 1 - ID:0040 - 0040 now stays up late at night gaming and has changed the router address so that he can continue gaming when his parents try to shut it off.*
*Prompt 2 - ID:0068 - At times, he has become physically and verbally aggressive due to his rigidity and inability to regulate emotions and behaviors when detaching from the screen.*
*Prompt 1 - ID:0037 - When parents stop him gaming, he video binges on YouTube, watching others play game all day.*
*Prompts 1 and 2 - ID:0043 - 0043 has gotten up in the middle of the night to hack into dad’s phone and then stays up all night gaming on it.*
*Prompt 1 - ID:0172 - He sneaks devices at night and plays when others are sleeping.*
*Prompt 2 - ID:0066 - When he must stop playing, he turns to videos of others playing, videos that often feature “foul” language and players who say mean things.*
*Prompt 2 - ID:0131 - Family is concerned for escalating altercations. Per 0131, when mom asks him to quit, he is usually able to end within 15–30 min. However, dad will frequently get home from work, storm up into his room and start yelling while 0131 is online with friends. ... Dad does not let him wrap up the session and will start pulling cords, which initiates conflicts.*
*Prompt 2 - ID:0070 - 0070 and his parents have gotten into a counterproductive feedback loop in which they distrust him, surveil him with software, and ground him for “bad behavior”. Resenting being “spied on”, 0070 rebels against their attempts to control and direct his online usage and tries to hack the system and continue his unfettered online behavior. To date, they have caught him every time, but both continue the cycle.*
*Prompt 2 - ID:0143 - Explosiveness has developed as 0143 has entered into adolescence. Parents report that the majority of his explosions occur in the context of limits around electronic media use.*
*Prompt 2 - ID:0198 - When mom caught him sneaking a game on his phone, argument escalated. Both mom and 0198 got emotional. 0198 was sobbing, which is unusual for him, saying he wants to stop, but feels like he cannot.*

Collectively, this dynamic appeared to represent a recurring, reactionary cycle (i.e., *evocation* with media) between caregivers and the child, stemming from *modulation* attempts to limit and then regain access to preferred media. Given sufficient time, media use is typically resumed in some flavor or form, if and when restricted via *creation*, *selection*, or *modulation*. While patients often used media as a primary emotion regulation strategy, their parents often defaulted to modulate their access to media as a solution to a *perception/appraisal* of harm *in situ*, prompting (at times) subsequent evocation—i.e., escalation and/or expressed distress.

**PCPOT**. As illustrated above, select *situational transitions with media* may have initiated, maintained, and/or escalated problematic use—particularly by progressively undermining a person’s psychosocial functioning via the occurrence and/or recurrence of select intrapersonal/interpersonal dynamics. The person-centric, case-by-case nature of clinical care necessitated due consideration of a given person (i.e., individual susceptibilities) (P—Person) to examine the spaces contextualizing their lived experience (C—Context), like the family microsystem (see **Figure 3**). Consideration of a person in context enabled one to determine the recurring processes (e.g., bio-psychological, social, and proximal) (P—Process) impacting them across select outcomes (O—Outcome) and their development/psychosocial functioning—i.e., the accumulation of outcomes over time (T—Time) (4, 5, 26, 36) (see **Figure 2**). Dispositional, developmental, and socio-contextual susceptibility factors can intermediate vulnerability to media effects (29).

## Discussion

The adoption and use of media from childhood into adolescence coincides with an array of impactful *transitions* (e.g., puberty, changing schools, moving, parental separation, and getting a job) and changing *bio-psycho-social* dynamics (e.g., changes in social cognition resulting from brain development), directly or indirectly related to a young person’s use of media in ways that can potentially initiate, maintain, or exacerbate PMU dynamics. Since many different types of *transitions* can lead to psychosocial problems (24, 39, 40), caution is warranted in pathologizing a patient’s media use in general. Rather, close examination of potentially hazardous media use dynamics is necessitated (15). Based on our research synthesis and results, PMU represents an experiential cycle of media-related dysfunction underpinned by select *situational transitions with media* as potential media use hazards. Given a young person (P—Person) and their immediate environmental context (C—Context), these *situational transitions with media* operate as pathways (P—Processes) with the potential to initiate and compound psychosocial impairment (O—Outcomes)—particularly given their recurrence over time (T—Time). Consideration of factors falling outside IGD/GD criteria is necessitated in practice to adequately address PMU, irrespective of whether clinical thresholds for IGD/GD are met.

### Person–context–process–outcome–time model of problematic media use

Several key insights emerged as a by-product of our qualitative findings and research synthesis when paired with extant work, like the I-PACE model (4) and IT-CPU (5). To better integrate and supplement each account, we proposed the PC-POT model of PMU (a needed person-centric reformulation of the mnemonic presented by Bronfenbrenner’s PPCT model from which the IT-CPU was derived) (5, 36). We believe that the mnemonic serves as a complementary basis to examine for the presence of potentially hazardous media use dynamics within the bounds of cognitive–behavioral, dialectic, and family therapy (for a review of treatment approaches, see 1, 44).

PC-POT also operates as a heuristic framework to support research more generally in this domain (e.g., assessment, adaptation, and refinement) in line with the National Institute of Mental Health’s RDoC Framework (30). PC-POT functions as a person- and process-centric, meta-theoretic framework that encompasses primary maintenance pathways proposed by established accounts of PMU (4, 5); it also uses concepts compatible with emotion regulation and situation research to facilitate further theoretical refinement and integration (see 8, 41, 42).

PC-POT suggests that *media use* can trigger specific interactions between people (P—Person) in specific situations (C—Context), leading to circumstantial changes (i.e., bio-psycho and socio-contextual) that establish or maintain PMU via select *situational transitions with media* (P—Process): *creation*, *selection*, *modulation*, *construal*, and *evocation* (see **Figures 2**, **3**). Each *situational transition with media* varies in their level of intentionality and effort (41), suggesting the existence of a general bias towards select transitions (see darker arrows **Figure 2**). The stipulation is also supported by established associations between select emotion regulation strategies (e.g., avoidance) and mental health impairments (8). As a result, rigidity in behavior and the recurrence of select transitions appear to exacerbate psychosocial impairments over time provided sufficient individual susceptibility, spanning select dispositional, developmental, and socio-contextual factors (see **Figure 2**) (1, 4, 5, 16). Because of the retrospective nature of our data, we were unable to assess these dynamics directly.

### Incentive sensitization and executive functioning: a lack of balanced use

Given sufficient time for habituation of media use, select media use activities may progressively become increasingly “automatic” as a preferred activity and associated with a broader array of internal and external cues over time (i.e., triggers promoting behavioral enactment) (4, 26); this makes facets of executive functioning (e.g., *inhibitory control*) and *incentive sensitization* paramount in assessing PMU dynamics (4, 45). Per our thematic review, however, the pathways outlined by I-PACE often related to psychosocial impairment via displacement effects resulting from *selection* as preferential media use, spanning each functional impairment domain. The pathways outlined by I-PACE—*selection* (e.g., as preferential media use), *modulation* (e.g., an increased amount of use), and *perception/appraisal* (e.g., as incentive sensitization and habituation of media-centric coping) (4)—appear to complete a self-reinforcing cycle that can eventually promote and compound psychosocial impairment (see Child pathways, **Figure 2**). However, such a self-reinforcing cycle largely presumes a lack of external *modulation* of *media availability/accessibility*. In this way, socio-contextual susceptibilities (e.g., lack of caregiver involvement) interact with other individual susceptibilities (e.g., dispositional or developmental factors that are associated with limitations in executive functioning) to promote PMU dynamics (see **Figure 2**) (4, 5). As documented, patients and caregivers both commonly contributed to PMU dynamics.

### Media-centric emotion regulation and compounding situational stress

The failure to address *situational stressors* directly (e.g., selection as avoidance) may indirectly maintain the experience of subsequent or ongoing stress to further catalyze problematic use (4, 46). Media use may serve as a knee-jerk response to manage *negative affective states* once habituated (4) and/or socialized (5). Reward learning may direct more immediate and reactive emotion regulation strategies to guide decision making under situations of stress, pressure, and/or urgency (e.g., emotion regulation as a model-free process) (31, 45–47). Progressive changes to one’s *perception/appraisal* of media activities (i.e., habituation of media-centric coping) is also conditioned over time via negative reinforcement (4, 5, 31, 46).

Instances wherein patients exhibited adjustment issues, like an overreliance on media-centric coping, *accentuation*—defaulting towards existing coping strategies/behaviors (40)—appeared commonplace. Patient charts clearly outlined that youth may experience an array of negative events, ranging from normative transitions to death of a loved one, that can challenge their capacity to adaptively cope. Given established media habits, patients tended to default towards media use as a conditioned tool for them to manage *negative affective states* in ways that stabilized (i.e., *selection* as preferential media use) or intensified their media use activities (i.e., *modulation* as increased engagement) (4, 12). These pathways are illustrated in **Figure 2** where an *in situ perception/appraisal* of media promotes subsequent media use as a coping strategy (see Child pathways).

### Caregiver involvement and perception/appraisal of media

Patients’ experience of PMU appeared to partially stem from caregivers’ involvement, which spanned a parallel set of situational transitions with media. For instance, a caregiver may manage a temperamental child by providing access to preferred media (i.e., *modulation*) to pacify their child in the moment (i.e., *selection* as avoidance) and avoid stress; both behaviors (i.e., permissive parenting towards child's media use, selection as avoidance) likely promote problematic use and remained conditioned via negative reinforcement over time (5). In clinic, caregivers (among other family members) primarily exhibited *selection* as avoidance (whether inferred or explicitly documented), likely due to the older age demographic of patients generally. Both caregiver *modulation* of *media access/availability* and caregiver *selection* (as avoidance) could, therefore, theoretically support PMU as an experienced cycle of media-dependent dysfunction (see **Figure 2**, Caregiver Pathways).

### Perception/appraisal as harm judgments and modulation of media availability

Caregiver *modulation* of *media access/availability* often served as a blunt intervention approach commonly employed to manage *perceptions/appraisals* of media-dependent harms. After all, if caregivers regard their child’s use of media as the cause of their suffering, they may seek to curtail their access to media directly. Harm judgments often occur quickly, remain based on attributions around the source of suffering, and can inform on how people evaluate a behavior (for a framework, see 18).

Restricting *media accessibility/availability*, however, appeared to promote a further escalation of PMU dynamics among patients seen in the clinic. As media is well ingrained in modern society (16), media use at a later point in time was typically resumed by the patient when restricted, whether permitted by a caregiver or not, and the cycle of dysfunction continued or escalated. Perhaps this is unsurprising as *incentive sensitization* predisposes individuals to seek out their given fixation (e.g., preferred media use) (31, 45) and may eventually come to rely on such as a coping tool a coping tool (4). Adolescents who regard parental rules around media use as overly restrictive reported higher levels of media overuse (48), suggesting that bluntly limiting *media access/availability* is far from a panacea (i.e., akin to a lack of caregiver involvement).

### An escalatory cycle of oppositional behavior

Patients commonly regarded caregiver interventions as harmful themselves and viewed their media positively (e.g., tool to meet normative goals, like socialization, or as way to cope), leading them to bypass media restrictions via *selection* (e.g., disregard of rules), *modulation* (e.g., watching videos of gaming when access to gaming was restricted), and/or *creation* (e.g., stealing a device). *Evocation*, as linkages between child and caregiver pathways (see **Figure 2**), mirrors oppositional behavioral dynamics in ways that support problematic use (5) and may account for several forms of psychosocial impairment seen across patients in the clinic.

Oppositional caregiver–child interactions can self-reinforce adverse interaction dynamics over time, harm the parent–child relationship, and impair a child’s psychosocial functioning (see 49). Assessing such dynamics in the context of PC-POT would align with calls to tie together *bio-psychological* processes with *social* interaction dynamics by the RDoC framework to explain core outcomes, like social attachment and psychosocial impairment (30). For example, among patients seen in the clinic, aggression/oppositional behavior commonly emerged as a reactive counterbalance to caregiver *modulation* attempts of *media access/availability*. When preferred media activities were restricted among patients, engagement often shifted to alternative media activities that remained accessible and/or related to preferred media (e.g., watching videos of Fortnite), not necessarily limiting the scope of activity by media type (e.g., gaming).

In line with escalatory dynamics characterizing oppositional behavior (49), PMU dynamics outlined by PC-POT may promote periods of social withdrawal (i.e., internalizing behaviors); coincide with an eventual resumption of the desired, yet now oppositional behavior; promote conflict/aggression (i.e., externalizing behaviors); and (given sufficient time) undermine caregiver–child *social attachment* as interpersonal relationships increasingly become colored by negative social interactions (i.e., interpersonal functioning impairments). The emergence of oppositional behavior stemming from caregiver *modulation* of *media accessibility/availability*, thus, helps to account for core longitudinal determinants of IGD, like loneliness and aggression (35).

### Assessment and treatment implications

Dispositional and developmental susceptibility. As the interaction between person (P—Person) in context (C—Context) (e.g., media environment/design and caregiver–child interaction dynamics) (4, 5) inherently governs whether a given young person will experience select situational transitions with media (P—Process) to the extent of experiencing impairment (O—Outcome) over time (T—Time), attention is necessitated towards assessing potential *dispositional* and *developmental susceptibilities* associated with executive functioning limitations (e.g., level of development and ADHD) to unpack “compulsive” modes of behavior, like PMU (4, 5, 16, 22). Most patients at presentation (99%, *n* = 203) exhibited some degree of impairment upon clinical assessment, with most patients exhibiting either dispositional (e.g., ADHD, ASD, anxiety, and depression) or developmental susceptibility (e.g., were a child or adolescent). Thus, treating underlying comorbidities represents a needed step to mitigate PMU dynamics and helping youth establish appropriate boundaries (e.g., *modulation* of amount, type, and/or context; *creation* of new media use situations) with their preferred media activities is also often necessitated to help youth learn to effectively downregulate media use impulses (16, 44).

Media-centric coping: emotion regulation. While media use may temporarily alleviate *negative affective states* as to reinforce further media use through intraindividual processes (i.e., negative reinforcement) (4, 5), circumstantial demands often warrant alternative courses of behavior to directly resolve forms of experienced stress/adversity. The degrees of freedom afforded by continually selecting media as a tool to disassociate from experienced *negative affective states* (*selection* as avoidance) is inherently constrained and may set the groundwork for oppositional behavior when *media access/availability* is restricted in due or undue ways abruptly (i.e., caregiver *modulation*).

As avoidance, rumination, and suppression represent common emotion regulation strategies employed by individuals suffering from mental health problems (8), supporting other emotion regulation strategies (e.g., cognitive reappraisal and problem solving) can help to address PMU dynamics via the bounds of conventional therapeutic approaches (e.g., CBT and DBT) (1, 16, 44). Meta-analytic results demonstrated that *dissociation* positively correlated with *disengagement* and *aversive cognitive perseveration* as emotion regulation strategies, but remained unrelated with more *adaptive emotion regulation strategies* (e.g., mindfulness, acceptance, and problem solving) (50). The adaptiveness of select emotion regulation strategies remain context dependent, however (8). Selection as avoidance (e.g., watching a funny movie or video > ruminating), for example, can adhere with distress tolerance skill building advanced by DBT (see (51)).

Socio-contextual susceptibility: past and present. *Perturbations*—or temporary changes in psychosocial functioning/behavior—occur for most youth due to circumstantial transitions (40) and may coincide with increased engagement with media (i.e., *selection* and *modulation*). Evaluations around *goodness-of-fit*, or the extent to which current circumstances meet the psychosocial needs of the child, and potential *cumulative events* (i.e., undergoing a series of stressful experiences) (40) warrant attention and may help to account for concurrent media engagement (4, 5).

*Timing effects* of select transitions can also predispose psychosocial impairment (40) and play a core role in the onset of mental health issues—especially during childhood and adolescence as vulnerable developmental periods (24). Both impaired *inhibitory control* and the routine experience of *negative affective states* may result from or coincide with experienced stressors and adversity within the scope of early development. The experience of deprivation and threat during childhood and adolescence is associated with declines in executive functioning (e.g., *inhibitory control* and *working memory*); differences in the magnitude of effect have been evidenced, indexing a stronger impact of deprivation on select executive functioning domains (see (52)). Conditioning of the brain’s threat system is front and center in child and adolescent development (24), outlining the need for early assessment and monitoring of *adverse childhood experiences* and recurring *situational stressors* in the context of PMU as individual susceptibility factors (see **Figure 2**).

Caregiver–child dynamics. Family therapy is often appropriate in most circumstances (1, 44, 53). Caregiver involvement may be necessary to help support the child’s balanced media use provided sufficient individual susceptibility or the presence of caregiver-dependent media use hazards. Attention should be placed on helping caregivers navigate developmentally appropriate, autonomy supportive supervision of their child’s media use, as well as help the child co-negotiate and establish healthy boundaries of use with their caregivers (44, 53). Presumably, caregivers sought clinical care due to a perceived insufficiency to effectively manage the situation on their own. If caregivers exhibit low levels of parental self-efficacy, they may default to non-optimal situational transitions with media as to facilitate a child’s media use (5), like *selection* as avoidance or *modulation* (as to excessively permit or bluntly restrict media use); extremes (e.g., micromanagement and overly permissive use) remain common among caregivers with children exhibiting problematic use (44).

Helping caregivers adjust their child’s media use activities in more nuanced ways (e.g., *creation* of new media use situations; *modulation* of type, amount, of context of use) could represent one approach to help mitigate such escalatory PMU dynamics (16, 44). Meta-analytic results demonstrated that *authoritative parenting* (e.g., warm parenting with clear boundaries/expectations), in addition to *active* (e.g., talking with your child about their use of media) and *restrictive mediation* (e.g., restricting or limiting access to media), can operate as caregiver strategies/approaches to reduce PMU (54–56). Restrictive mediation (e.g., limiting *media access/availability*), while potentially efficacious among younger children, appears to diminish in its utility as a strategy to mitigate the experience of problematic use during adolescence (55).

Two patients (< 1%, *n* = 2) did not exhibit functional impairment across any domain per their clinical assessment. This group may reflect a larger portion of the population who feel that their child is “addicted” to using media or view it as “problematic” due to oppositional behavior, perceived inappropriate, or excessive use—but may not necessarily seek treatment or exhibit severe forms of impairment warranting clinical care. Time spent using media itself is a non-sequitur for determining whether a child has a media use problem (i.e., GD/IGD) (14). 

Perceptions of harm can also vary by person and culturally (17, 18), with caregiver concerns around media spanning an array of activities (e.g., watching porn or aggressive content, sexting, and distractibility/declining academic performance) at patient presentation. Caregiver *perceptions/appraisals* of media-dependent harm may assume causality in ways that could exacerbate actualized harm by attributing suffering to media use specifically and independently of other factors (53), effectively setting the stage to potentially catalyze escalatory PMU dynamics. Addressing parental/caregiver perceptions/appraisals around a child’s media use, therefore, represents a critical intervention step, and may operate as a treatment strategy to mitigate the establishment or propagation of escalatory PMU dynamics. As care/harm violations appear to underpin moral judgments generally (e.g., fairness and purity) (18), different value systems/beliefs among caregivers likely account for changes in caregiver concerns (i.e., *perception/appraisal* as harm judgments) around their child’s media use (for a framework, see (18)).

Digital careerism as an example. Socio-economic factors may promote compulsive behavior by reducing “exploration” and driving narrowed “seeking” behaviors in some circumstances (17). Both Iranian and Chinese youth experiencing IGD/GD express interest in a digital career (e.g., influencer, creator, and pro-gamer) (57, 58). Similar media-related, occupational pursuits were also noted in the present sample. For select youth experiencing IGD/GD, media use appeared to be partially directed at engaging in a gratifying, feasible, and potentially lucrative career path marked by multiple young, yet visible exemplars (e.g., e-sports gamers) (57, 58). Social learning theory, after all, helps to partially explain the maintenance of PMU (5).

Irrespective of cultural differences, caregivers may disregard digital careers as an illegitimate choice of vocation (58), with excessive media use often raising concerns among caregivers around diminished academic performance. As noted by the present sample, academic demands may promote experiences of stress on the part of the child and prompt negative caregiver–child interactions stemming from caregiver concerns around educational/vocational functioning, *selection* as avoidance on the part of the child to cope, and potential oppositional dynamics when *media accessibility/availability* is restricted. Comparable dynamics were also noted among rural Chinese (57) and Iranian youth exhibiting problematic use (i.e., IGD/GD) (58).

Active mediation could potentially help caregivers and children bridge media-based disagreements. Youth struggling with media-related problems may consider viable, yet alternative career paths related to media use (58). Broadening the scope of potential career paths considered by both parties (e.g., videographer, independent journalist, and programmer) may help reduce tensions around preferred digital activities by promoting collaborate problem solving (e.g., creation, modulation) or help to balance negative parental/caregiver *perceptions/appraisals* of their child’s media activities by identifying inlets to support skill building (53).

Lastly, evidence suggests that child *perceptions/appraisals* of their parent’s media use rules (e.g., personal adherence to the rules themselves and perceived restrictiveness) may affect media overuse; helping youth and parents find common ground to facilitate balanced use and co-established media use rules (regarded as fair by both parties) likely represents a necessary step to help mitigate media overuse (48); encouraging engagement in an offline leisure activity and modeling of balanced use are recommended (44).

### Limitations and future directions

Despite the strengths of the present study, it maintains key limitations. We conducted a mixed-methods analysis using generative AI (i.e., ChatGPT-4o) to capitalize on the strengths of the technology (e.g., explicit prompts to extract relevant data from qualitative text, reproducibility) and enable others to apply, iterate on, and ideally reproduce aspects of this work via shared prompts and AI models (as unitized forms of social knowledge). Owing to the overlap between functional domains, it remained outside the scope of the present study to confirm the validity of each individual excerpt by domain manually. Excerpts were reviewed by classification domain (e.g., sustainable functional autonomy, interpersonal functioning, etc.) to quantify impairment frequencies using human coders. Excerpts were then selectively extracted to capture the breadth of impairments exhibited at patient presentation to support our qualitative analysis. Maximizing AI sourced excerpts for domain validity would represent an area of future research. Moreover, the core pathways outlined by PC-POT remain based on data from a sample of youth accessing care for media use problems and warrant validation across alternative samples. PC-POT remains based on extant work (e.g 4–6, 8, 18, 36, 40–42, 49), suggesting that the insights generated may transfer to other samples; specific adaptations to the framework are also encouraged. PC-POT stresses attention towards individual-level susceptibility factors potentially underpinning a given case of PMU; situational transitions with media afford a flexible way to analyze PMU considering such factors. Lastly, because of the nature of patient data, assessment of media environments/design remained limited. The substance of youths’ media environment and media design warrants due consideration in the onset and escalation of problematic use (4, 5).

## Conclusion

The PC-POT framework complements existing accounts of problematic use by offering a media-agnostic, person- and process-centric account of PMU rooted within the context of youths’ lived experience (i.e., PMU dynamics as an experiential cycle, spanning select bio-psycho-social processes). We hope that this work encourages customized application and adaptations of PC-POT, in addition to the methods employed (i.e., AI prompts), to further systematize research and clinical practice on this topic for the benefit of all youth. PC-POT is meant to function as a flexible, heuristic theoretical account to support inquiries around potential forms of media-dependent harm. 

## Data Availability

The datasets for this article are not publicly available due to concerns regarding participant/patient anonymity. Requests to access the datasets should be directed to the corresponding author.

## Footnotes

1 The case was modeled after 0134, with added context integrated from other patient charts (e.g., COVID and personal views about their media use); the summary represents an abbreviated, exemplar case example. Elements of 0314’s chart were excluded from the summary for brevity (e.g., autism spectrum disorder, anxiety, and oppositional defiant disorder diagnoses). EN is an arbitrary pseudonym.

2 Phase 1 coding took place among a subset of charts (n = 200) and included patients classified for clinically significant levels of media-related problems, irrespective of media type. Classifications were made per clinician judgment.
