# Supplementary material for: Placing problematic media use in context: a research synthesis, person-centric framework, and chart review among a clinical sample of US youth
Source: Front Psychiatry. 2025 Sep 30;16:1574502. doi: 10.3389/fpsyt.2025.1574502 (PMC12519947; doi:10.3389/fpsyt.2025.1574502)
Supplement: Supplementary file 1 [file DataSheet1.docx]

**SUPPLEMENTARY MATERIALS**

**Prompt Engineering: Generative AI.** Prompts were iteratively created and refined based on use guidance around generative AI in healthcare settings (provided by internal training at the primary author’s institution). The prompt engineering recommendations included outlining a primary role, instructions, relevant context, output constraints, and examples in the prompt (i.e., RICCE), in addition to leveraging “chain-of-thought” reasoning. Early versions of the prompt were provided to ChatGPT (e.g., to reformat the desired task into a “chain-of-thought” format, for feedback to mitigate the odds of hallucinations) for further fine-tuning. Definitions of each functional domain were also initially sourced from ChatGPT and further refined by the clinical portion of our research team. Lastly, we split our final prompt into two prompts; each had a separate core task (i.e., classification of functional impairment with examples of hazardous media use dynamics, sourcing excerpts encompassing hazardous media use dynamics for positive impairment classifications by domain without examples). The dual prompt approach helped to maximize outputted data for subsequent qualitative review and simplify prompt complexity. Each prompt itself followed a sequential “chain-of-thought” approach via a series of steps to guide the extraction of relevant patient information for subsequent review; the AI was instructed to output its results in JSON data format.

**Prompt 1.** The prompting process first (1) provided an overview of the task (**Role**, **Instruction**), and (2) then definitions for each functional domain (**Context**). Next, it (3) had the AI identify and extract excerpts related to a patient's functioning by domain, (4) classify whether a given domain exhibits impairment per the documentation provided, and (5) to output excerpts supporting positive impairment classifications made by domain. A final series of “checklist” steps were provided per ChatGPT’s recommendation (**Constraints**), along with an example template output (**Example**). Examples of common hazardous media use dynamics noted in clinic were included in the template example to encourage their inclusion in the AI output without restricting output to only media-related examples. This helped to ensure that extracted excerpts highlighted excerpts pertinent to the patient’s functioning for manual validation.

**Prompt 2.** Prompt 2 built off and directly complemented Prompt 1. Its prompting process was identical to Prompt 1 (steps 1-2). However, steps 3 through 5 were explicitly tied to identifying excerpts related to the patient’s media use and did not include step 4. For each patient chart, we piped in the classification matrix output from Prompt 1 into Prompt 2 as the basis for sourcing supporting excerpts encompassing potential hazardous media use dynamics for each impairment domain classified as present, if identified by the AI in the patient chart. In contrast to Prompt 1, the template was absent of any concrete examples to mitigate bias in outputted excerpts. Due to the tailored nature of Prompt 2, the template example also included the chart’s study identification number for documentation purposes.

**Excerpts.** Extracted qualitative content was restricted to excerpts to support classifications of impairment by domain, with an emphasis on including documented associations related to the patient’s use of media by impairment domain explicitly in the secondary prompt. The primary prompt yielded 2,439 excerpts across the five functional domains in total. The secondary, supplementary prompt yielded 2,397 excerpts across the five functional domains in total for review (*n* = 948 duplicate excerpts were identified when cross-checking excerpts within each domain between Prompt 1 and 2, 39.55%).

**Hallucinations and Excerpt Validation.** Several steps were taken to ensure data quality and mitigate known issues with the use of generative AI systems (i.e., hallucinations). This included including specific language (e.g., unedited excerpts) in the prompt, repetition (e.g., unaltered, unedited, etc. excerpts), adding in an explicit validation step (e.g., cross-check all excerpts to ensure they are verbatim), and checking each excerpt outputted.

All excerpts extracted from patient charts reported in our results were also checked via an automated pipeline in python. We leveraged the *partial_ratio()* function from the package, *TheFuzz* (version 0.22.1), to identify excerpts matching the AI output due to its capacity to match partial phrases;^[[1]](#footnote-1)^ the function leverages Levenshtein Distance—a metric indexing the number of single character edits, deletions, or substitutions needed to match two strings—to calculate a similarity score (Mugaanyi et al., 2024).^[[2]](#footnote-2)^ The score was calculated between AI extracted excerpts with text excerpts directly sourced from the patient chart using a sliding window approach. The *python-levenshtein* python module (version 0.26.1) was used to calculate Levenshtein Distance between the best candidate excerpt (identified by the highest partial ratio score for each candidate excerpt) and the AI outputted excerpt to demonstrate the veracity of the validation approach.^[[3]](#footnote-3)^ An adjusted Levenshtein distance score was used to better approximate partial matches between the AI output and the best candidate excerpt—particularly when superficial changes to punctuation (e.g., don’t, dont) or whitespace (e.g., deleting extra whitespace) were made. This entailed removing all non-alphanumeric characters, extra spaces, and case sensitivity to match candidate excerpts with the AI output using the *re* function, *findall()*, and calculating Levenshtein distance on *alphanumeric sequences*.

In cases wherein a perfect alphanumeric match was not identified (11.08%, *n* = 536), it implicated that the *content* of the text was potentially edited by the AI during the extraction process (i.e., content alteration or hallucination). These entries were manually screened by the primary author and checked by a research assistant. The vast majority of these cases included instances wherein the AI output deleted whole words or phrases (*n* = 107, 19.96%); altered spelling mistakes and/or inserted words or phrases (e.g., …a mountain, …mountain; …the dinner table, …dinner table) to form a complete statement or provide added context (e.g., had trouble [paying attention] b/c of ADHD…) (*n* = 223, 41.61%); swapped terms (e.g., “patient”, “patient id”, “pronouns”) (*n* = 124, 23.13%); or paraphrased/fabricated information (*n* = 26, 4.85%). A small number of entries also included punctuation errors in the chart documentation (e.g., comma preceding a period) which impaired our ability to automatically match the AI excerpt with the chart text (*n* = 10, 1.87%). A number of entries also spanned more than one type of alteration across the aforementioned categories (*n* = 46, 8.58%). In all, these results suggested that the odds of outright fabrication of chart content was largely negligible, but nevertheless warranted attention. Manual screening of identified fabrications/paraphrased excerpts infrequently misrepresented the substance of the chart itself when reviewed. These entries largely reflected issues with “who reported what” (e.g., saying the “patient” said something that a “parent” reported), the directionality (i.e.,  increased → decreased), or other clerical mistakes (e.g., altering the patient’s chart id). In all, these shortcomings remain easily addressable via improving clinician documentation (per Chat GPT’s recommendation) using things like AI-based tools (which was beyond the scope of the present study) and employing a screening procedure to verify AI output.


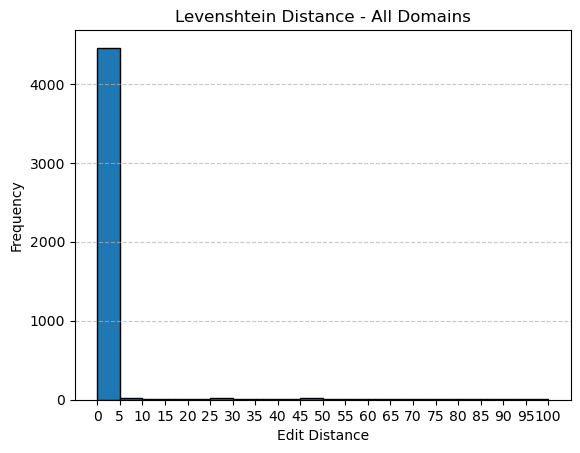


Figure S1. *Notes.* Levenshtein distance scores are based on alphanumeric sequences, except when an identical match was not found. In such cases, scores represent values based on the candidate excerpts and AI generated excerpts.


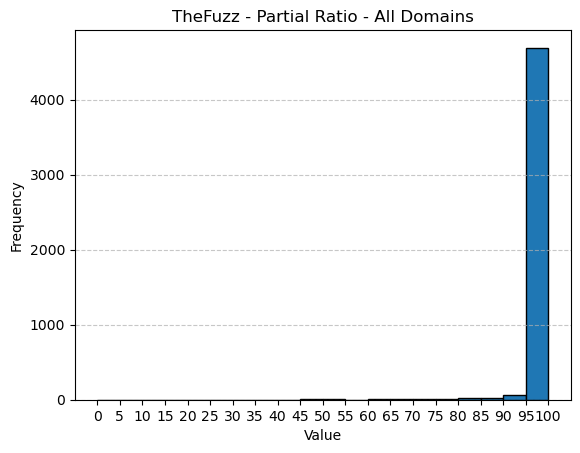


Figure S2. Partial Ratio Scores. *Notes.* Values represent similarity scores between candidate excerpts and AI generated excerpts.

**Human Coding.** All AI classifications made were screened by two human coders (see steps below). All excerpts derived from Prompt 1 and 2 were presented to coders in a Word document for this final validation step; excerpts were highlighted to index whether they represented a perfect (Green) or partial match (Yellow). Levenshtein distance and partial ratio scores were also provided to supplement their review as confidence indicators. For partial matches, if the candidate excerpts did not include the AI extracted phrase, coders were instructed to manually check the patient chart. Lastly, human coders were also instructed to source additional excerpts in instances of false negative classifications, or true positive classifications with inadequate support.

To streamline the coding process, coders were instructed to edit the AI classifications; edited corrections were used to establish inter-coder reliability (1 = corrected, 0 = non-edited). A random sample of data (*n* = 100, approx. 50% of all charts for functional impairment classifications) served as the basis for calculating inter-coder reliability due to the high level of accuracy across the AI classifications. Coders demonstrated a sufficient level of agreement across the reliability set, irrespective of domain (α = Krippendorff’s Alpha): sustainable functional autonomy (α = .77), externalizing behaviors (α = 1.0), internalizing behaviors (α = .79), educational/vocational functioning (α = .81), and interpersonal functioning (α = .76). The coding set was resolved by randomly selecting entries between coders and the remaining charts split equally between coders for independent coding.

**CODEBOOK AND PROMPTS**

**Prompt 1 (Domain-Centric Instructions, Media Examples)**

Imagine you are a behavioral health clinician. The goal of this task is to classify patient charts based on the patient’s exhibited functional impairment by domain and to document supporting evidence for these classifications using unedited excerpts from the patient chart. The final output should be in JSON format.

1. **Understand Functional Domains**

- ***Externalizing Behaviors**:* Outward actions impacting oneself, others, or the environment, often characterized by aggression, impulsivity, disruptive conduct, self-harm, or delinquency.

- ***Internalizing Behaviors**:* Inward emotional struggles (e.g., depression, anxiety, somatic complaints), often associated with heightened distress, withdrawal, or impaired self-regulation.

- ***Interpersonal Functioning**:* The ability to establish, maintain, and navigate social relationships effectively, characterized by communication skills, empathy, conflict resolution, and reciprocity.

- ***Educational/Vocational Functioning**:* Performance and adaptability within structured academic or workplace environments, including task management, problem-solving, and achievement relative to developmental expectations.

- ***Sustainable Functional Autonomy**:* The capacity to independently perform daily living tasks (e.g., sleep, personal hygiene, diet) and maintain physical, emotional, and social well-being over time, adapting to life changes and stressors without excessive reliance on external support.

2. **Analyze Patient Chart**

- Identify and extract unaltered excerpts related to any of the functional domains above.

3. **Classify and Document**

- For each patient chart: *Create a matrix* indexing the types of functional problems exhibited by domain (1 = Present, 0 = Absent).

- For each positive classification by domain (1 = Present): Extract *supporting excerpts* from the patient chart. Supporting excerpts should: (1) include unaltered quotes about the patient used to make the classification and (2) quote complete sentences whenever possible.

4. **Verify Checklist and Format**

- Cross-check all excerpts to ensure they are verbatim, with no alterations, omissions, or paraphrasing.

- Ensure all positive classifications are supported by direct evidence in sourced excerpts.

- Recode unsupported or speculative classifications.

- Output an empty list for “supporting_excerpts” if a given functional domain is classified as absent (0).

- Use the following JSON format:

{ "patient_id": "0000", "functional_impairment_matrix": { "externalizing_behaviors": 1, "internalizing_behaviors": 1, "interpersonal_functioning": 1, "educational_vocational_functioning": 1, "sustainable_functional_autonomy": 1 }, "impairment_details": [

{"domain": "externalizing_behaviors", "supporting_excerpts": ["When access to media is limited, 0000 gets aggressive, makes threats, and has broken things.", "They sneak in screen time at night when media access is restricted by parents.", “Family fights excessively about 0000’s media use.", "Parent states, 0000 stole money to make unapproved digital purchases."]},

{ "domain": "internalizing_behaviors", "supporting_excerpts": ["0000 reports relying on media to deal with their stress.", "Parent is concerned about depression themed content they view online.", "0000 is preoccupied and anxious about a virus on their phone.", "They have had their nudes leaked online. 0000 is worried and distressed about social backlash.", "0000 spends most of their time online and self-isolates in-person."]},

{ "domain": "interpersonal_functioning", "supporting_excerpts": ["Parent thinks 0000 spends too much time online and neglects their chores.”,  “Family fights excessively about 0000’s media use.", ‘0000 says they struggle socially and spend most of their free time at school alone on their phone."]},

{ "domain": "educational_vocational_functioning", "supporting_excerpts": ["Patient says they get distracted watching videos while trying to do schoolwork.", "Parent says patient’s grades have fallen over time.", "Patient is not motivated to engage in non-media activities."]},

{ "domain": "sustainable_functional_autonomy", "supporting_excerpts": ["0000 has a hard time waking and stays up late on social media messaging friends.", "0000 reports ongoing sleep disturbances.", "0000 skips showers and meals when spending long hours online."]}]}

- Validate the output for JSON format correctness.

**Prompt 2 (Media-Centric Instructions, No Examples)**

Imagine you are a behavioral health clinician. The goal of this task is to document supporting evidence for functional impairment classifications using unedited excerpts from the patient chart. The final output should be in JSON format.

1. **Understand Functional Domains**

- ***Externalizing Behaviors**:* Outward actions impacting oneself, others, or the environment, often characterized by aggression, impulsivity, disruptive conduct, self-harm, or delinquency.

- ***Internalizing Behaviors**:* Inward emotional struggles (e.g., depression, anxiety, somatic complaints), often associated with heightened distress, withdrawal, or impaired self-regulation.

- ***Interpersonal Functioning**:* The ability to establish, maintain, and navigate social relationships effectively, characterized by communication skills, empathy, conflict resolution, and reciprocity.

- ***Educational/Vocational Functioning**:* Performance and adaptability within structured academic or workplace environments, including task management, problem-solving, and achievement relative to developmental expectations.

- ***Sustainable Functional Autonomy**:* The capacity to independently perform daily living tasks (e.g., sleep, personal hygiene, diet) and maintain physical, emotional, and social well-being over time, adapting to life changes and stressors without excessive reliance on external support.

2. **Analyze Patient Chart**

- Identify and extract unaltered excerpts related to any of the functional domains above, focusing on information that also highlights how each domain may relate to the patient’s use of media (e.g., books, screen devices, social media, video games, pornography).

3. **Classify and Document**

- INSERT IMPAIRMENT MATRIX HERE

- For functional problems classified as (1 = Present) in the dictionary above: Extract *supporting excerpts*** from the patient chart. Ensure the supporting excerpts: (1) Include unaltered quotes from the patient chart that justify the classification, focusing on documented reports linking the impairment to the patient’s use of media. (2) Quote complete sentences whenever possible.

4. **Verify Checklist and Format**

- Cross-check all excerpts to ensure they are verbatim, with no alterations, omissions, or paraphrasing.

- Cross-check extracted excerpts with the patient’s chart to ensure all documented connections between impairments and media use are included.

- Ensure all positive classifications are supported by direct evidence in sourced excerpts.

- Recode unsupported or speculative classifications.

- Output an empty list for “supporting_excerpts” if a given functional domain is classified as absent (0) or if no reports linking the impairment to the patient’s use of media are documented.

- Use the following JSON format:

{ "patient_id": "0000",

"impairment_details": [

{ "domain": "externalizing_behaviors", "supporting_excerpts": ["...", "..."]},

{ "domain": "internalizing_behaviors", "supporting_excerpts": ["...", "...", "...", "..."]},

{ "domain": "interpersonal_functioning", "supporting_excerpts": ["...", "...", "..."]},

{ "domain": "educational_vocational_functioning", "supporting_excerpts": ["...", "...", "..."]},

{ "domain": "sustainable_functional_autonomy", "supporting_excerpts": ["...", "...", "...", "...", "..."]},

]}

- Validate the output for JSON format correctness.

**Prompt V11 (Example: Previous Iteration to Finalized Prompts)**

Imagine you are a behavioral health clinician. The goal of this task is to classify patient charts based on functional impairments, document supporting evidence for these classifications, and analyze the relationship between these impairments and media use. The final output should be in JSON format, summarizing the findings for each patient.

1. **Understand Functional Domains**

- ***Externalizing Behaviors**:* Outward actions impacting oneself, others, or the environment, often characterized by aggression, impulsivity, disruptive conduct, self-harm, or delinquency.

- ***Internalizing Behaviors**:* Inward emotional struggles (e.g., depression, anxiety, somatic complaints), often associated with heightened distress, withdrawal, or impaired self-regulation.

- ***Interpersonal Functioning**:* The ability to establish, maintain, and navigate social relationships effectively, characterized by communication skills, empathy, conflict resolution, and reciprocity.

- ***Educational/Vocational Functioning**:* Performance and adaptability within structured academic or workplace environments, including task management, problem-solving, and achievement relative to developmental expectations.

- ***Sustainable Functional Autonomy**:* The capacity to independently perform daily living tasks (e.g., sleep, personal hygiene, diet) and maintain physical, emotional, and social well-being over time, adapting to life changes and stressors without excessive reliance on external support.

2. **Analyze Patient Chart**

- Identify and extract unaltered excerpts related to any of the functional domains above, making sure to collect statements linking each domain to the patient's use of media (e.g., books, screen devices, social media, video games, pornography).

3. **Classify and Document**

- For each patient chart: *Create a matrix* indexing the types of functional problems exhibited by domain (1 = Present, 0 = Absent).

- For each positive classification by domain (1 = Present): Extract *supporting excerpts* from the patient chart. Supporting excerpts should: (1) include unaltered quotes about the patient used to make the classification, (2) illustrate the scope of impairment, and (3) document any explicit reports linking the impairment with the patient’s use of media. (4) Quote complete sentences when possible.

4. **Verify Checklist and Format**

- Ensure excerpts clearly contextualize the patient’s functional state.

- Cross-check extracted excerpts with the patient’s chart to ensure all documented connections between impairments and media use are included.

- Cross-check all excerpts to ensure they are verbatim, with no alterations, omissions, or paraphrasing.

- Ensure all positive classifications are supported by direct evidence in sourced excerpts.

- Recode unsupported or speculative classifications.

- Output an empty list for “supporting_excerpts” if a given functional domain is classified as absent (0).

- Use the following JSON format:

{ "patient_id": "0000",

"functional_impairment_matrix": { "externalizing_behaviors": 1, "internalizing_behaviors": 1, "interpersonal_functioning": 1, "educational_vocational_functioning": 1, "sustainable_functional_autonomy": 1 },

"impairment_details": [

{ "domain": "externalizing_behaviors", "supporting_excerpts": ["...", "..."]},

{ "domain": "internalizing_behaviors", "supporting_excerpts": ["...", "...", "...", "..."]},

{ "domain": "interpersonal_functioning", "supporting_excerpts": ["...", "...", "..."]},

{ "domain": "educational_vocational_functioning", "supporting_excerpts": ["...", "...", "..."]},

{ "domain": "sustainable_functional_autonomy", "supporting_excerpts": ["...", "...", "...", "...", "..."]},

]}

- Validate the output for JSON format correctness.

**Generative AI Prompt Evaluation Example (Prompt V11)**

**Query**

*If provided with patient charts, what are the odds (10 being guaranteed and 0 being impossible) that the prompt will lead chatgpt, as a generative AI system, to output fabricated information based on the language provided?*

**Output**

The odds of this prompt leading ChatGPT to output fabricated information depend on several factors, primarily how the patient charts are presented and whether the AI adheres to the constraints outlined in the prompt. Based on the language provided and the typical behavior of generative AI systems, the likelihood of fabrication can be estimated as follows:

**Key Considerations:**

1. **Explicit Constraints on Using Provided Data**:

   - The prompt explicitly states that supporting excerpts must come directly from the patient charts and prohibits speculative or unsupported classifications. This reduces the risk of fabrication.
2. **Generative Nature of the AI**:

   - While ChatGPT is designed to process and analyze provided data, it may occasionally generate plausible-sounding but fabricated content if:
     - The input data is ambiguous, incomplete, or lacks explicit information for required classifications.
     - The task involves extrapolation beyond the provided information.
   - However, if the AI is strictly instructed to output only what is explicitly stated in the charts (as this prompt specifies), the odds of fabrication decrease significantly.
3. **Risk Mitigation Steps in the Prompt**:

   - The requirement to cross-check excerpts with the original charts and to output an empty list for absent domains further reduces the risk of fabrication.
4. **Potential Gaps**:

   - If the AI misinterprets ambiguous data or fails to recognize insufficient evidence, it might inadvertently produce unsupported classifications or excerpts.
   - The JSON validation step ensures technical correctness but does not inherently verify the factual accuracy of the content.

**Estimated Odds:**

- **Score: 2/10 (Low Probability of Fabrication)**
  - The prompt's clear instructions and constraints significantly mitigate the risk of fabrication. However, there is a slight chance of error due to ambiguity in patient charts, misinterpretation, or the generative nature of the AI.

**Recommendations to Further Reduce Risk:**

1. **Reinforce Input Clarity**: Ensure patient charts are detailed, well-structured, and contain explicit evidence for all domains.
2. **Set Output Verification Steps**: Use additional tools or manual review to validate that all generated content aligns with the provided data.
3. **Emphasize Prohibition of Inference**: Reiterate that the AI should not infer or extrapolate information beyond the provided charts.

By following these measures, the risk of fabrication can be minimized even further.

**Generative AI Prompt Evaluation Example (Prompt V11)**

**Query**

*Rate the prompt on a scale from 1 (being worst) and 10 (being best) based on how it should perform as a prompt when using generative AI to process text data.*

**Output**

**Rating: 9/10**

The prompt is highly effective for using generative AI to process text data, particularly for structured tasks involving classification, evidence extraction, and JSON output formatting. Here's the breakdown:

**Strengths:**

1. **Clarity and Structure (10/10):**
   - The task is broken into logical steps with clear instructions, making it easy for the AI to follow.
   - Definitions for functional domains are explicit, reducing ambiguity.
2. **Specificity (9/10):**
   - The requirements for the output format (JSON structure) are well-defined, which ensures consistency in the results.
   - Explicit instructions to use direct quotes and to avoid speculative classifications help mitigate risks of fabrication.
3. **Error-Handling and Validation (9/10):**
   - The inclusion of a checklist and validation steps encourages accurate and reliable outputs.
   - The prompt accounts for cases where data may be absent, requiring an empty list for unsupported classifications.
4. **Adaptability to AI Capabilities (9/10):**
   - The task aligns well with the strengths of generative AI, such as text classification, summarization, and structured data output.

**Weaknesses:**

1. **Risk of Ambiguity (8/10):**
   - If patient charts contain vague or incomplete information, the AI might struggle to extract relevant evidence without additional guidance.
   - The prompt assumes the AI will always correctly identify and interpret explicit evidence, which could be challenging in edge cases.
2. **Potential Over-Specification (9/10):**
   - The detailed requirements, while useful, might overwhelm the AI in complex scenarios with extensive or conflicting data.

**Suggestions for Improvement:**

1. **Clarify Ambiguity Handling:**
   - Add guidance for handling ambiguous or conflicting information in patient charts.
   - Example: "If evidence for a classification is unclear, classify the domain as absent (0) and include a note explaining the ambiguity."
2. **Define JSON Validation:**
   - Specify whether the JSON output should be programmatically validated (e.g., schema checks) and how errors should be handled.
3. **Edge Case Instructions:**
   - Include examples or scenarios for edge cases, such as charts with missing or contradictory data, to further guide the AI.

**Final Thoughts:**

This prompt is robust and well-suited for the intended purpose. Minor refinements to address ambiguity and edge cases could elevate it to a perfect 10.

1. https://pypi.org/project/thefuzz/ [↑](#footnote-ref-1)
2. https://www.jmir.org/2024/1/e52935/ [↑](#footnote-ref-2)
3. https://pypi.org/project/python-Levenshtein/ [↑](#footnote-ref-3)
